# The association of the timing of outpatient palliative care clinic visit on the utilization of hospital services and decision making at the end of life in patients with cancer – a retrospective cohort study

**DOI:** 10.1186/s12885-025-15242-1

**Published:** 2025-11-18

**Authors:** Anu Anttonen, Timo Carpén, Nelli-Sofia Nåhls, Pauliina Kitti, Sofia Miinalainen, Riikka-Leena Leskelä, Outi Akrén, Tiina Saarto

**Affiliations:** 1 Department of Oncology, Comprehensive Cancer Center, Helsinki University Hospital and University of Helsinki, Helsinki, Finland; 2 Palliative Care Center, Comprehensive Cancer Center, Helsinki University Hospital, University of Helsinki, Helsinki, Finland; 3 Department of Oncology, Vaasa Central Hospital, The Wellbeing services County of Ostrobothnia, Vaasa, Finland; 4 Palliative Center, Turku University Hospital and University of Turku, Turku, Finland; 5 Nordic Healthcare Group, Espoo, Finland and University of Helsinki, Helsinki, Finland

**Keywords:** Palliative care, End of life, Cancer, Emergency department, Healthcare utilization

## Abstract

**Background:**

The timely integration of palliative care (PC) into cancer treatment improves quality of life and supports care planning at the end of life (EOL) for patients with incurable cancer. However, there is limited data on how the timing of outpatient PC clinic visits affect hospital services use and decision-making as death approaches. The study evaluated the association of outpatient PC clinic visit, and its timing, on healthcare use and decision making at the EOL in patients with cancer. Additionally, the implementation of the integrated PC was examined.

**Methods:**

Data on all patients (*n* = 3744) with cancer treated at the Comprehensive Cancer Center Helsinki University Hospital during 2017–2018 and deceased by the end of 2018 were retrospectively reviewed. The data on healthcare utilization was extracted from the hospital database. The timing of the outpatient PC clinic visit was determined as follows: (1) Integrated PC visit i.e. PC was initiated alongside the usual oncological care (2) Non-integrated PC visit i.e. the first visit occurred after the termination of disease modifying treatments, and (3) No visit.

**Results:**

In total, 2151 (57.4%) patients visited an outpatient PC clinic, and 1207 (32.2%) patients had an integrated PC visit. The median time from the first PC visit to death among integrated PC group was significantly longer compared to the non-integrated PC group (129 vs. 55 days; *p* < 0.001).

Patients with an integrated or a non-integrated PC visit were less likely to be hospitalized during the last 30 days of life compared to patients with no outpatient PC clinic visit (36.5% vs 43.6%; p<0.001 and 35.6% vs 43.6%; p<0.001, respectively). Patients with PC visits also had lower number of inpatient days. No significant differences were observed in emergency department visits between the groups (37.8%, 37.3% and 40.3%, respectively).

**Conclusions:**

Overall, access to tertiary center outpatient PC clinic was relatively high, as more than half of the patients visited the outpatient PC clinic. In one third the first visit was considered as integrated. However, the first integrated visit occurred only four months prior to death. Despite this, both integrated and non-integrated outpatient PC clinic visits suggested a possible association with lower hospitalization rates and fewer inpatient days at the EOL.

**Supplementary Information:**

The online version contains supplementary material available at 10.1186/s12885-025-15242-1.

## Background

Worldwide, cancer is a leading cause of death, accounting nearly one in six deaths and affecting millions of individuals and their families [1, 2]. According to the International Agency for Research on Cancer, the global burden is expected to rise to 21.6 million new cases and 11.3 million deaths by 2025 [3]. Cancer is associated with a wide variety of distress and impaired quality of life (QoL) as well as physical and psychological functioning [4]. Therefore, concurrently with active oncological treatment, it is important to also pay preventive attention to the patient’s psychosocial and physical functioning. The goal of palliative care (PC) is to relieve suffering caused by the disease and enhance the well-being of the patient and their families throughout the disease trajectory.

Early access to PC has been associated with improvement in QoL among patients with advanced cancer [5]. In addition, it is associated to less aggressive treatments at the end of life (EOL) [6–10]. Indeed, one of the key goals of PC is to reduce the need for acute hospital services at the EOL by offering sufficient support and advance care planning [11]. As PC has shown multiple benefits for patients with cancer, the integration of palliative and oncological care is now considered as standard care in the treatment of advanced cancer.

Astonishingly, despite this evidence, population-based studies of cancer care have shown an increase in the aggressiveness of treatment at EOL. Younger patients, males, those living in a rural area, patients with comorbidities and those with lung, breast or hematological malignancies are more likely to receive aggressive treatment [12, 13]. Naturally this leads to an increased need for hospital services and significantly increase healthcare costs [14]. Thus, even though clinicians and healthcare organizations are aware that early PC should be provided, its systematic implementation in clinical practice remains a challenge [15].

The aim of our study was to evaluate the access to PC, the implementation of the integrated PC as well as the association of an outpatient PC clinic visit and its timing on the utilization of hospital services and decision making at the EOL in patients with cancer.

## Methods

### Cohort selection

This retrospective study cohort comprised all adult (≥ 18 years) patients with cancer diagnosis (ICD-10 C00-C96) with at least one contact at the Comprehensive Cancer Center of the Helsinki University Hospital in 2017–2018 and deceased by the end of 2018. Patients who died between July 1, 2017, and December 31, 2018 (*n* = 3744), were included to ensure a look-back period of minimum 6 months prior to death.

In Finland, cancer treatment is mainly provided in secondary and tertiary hospitals. Helsinki University Hospital (HUH) is the largest of the five tertiary hospitals in Finland being responsible for the cancer care of a population of approximately 1.7 million in Southern Finland. During the time of this study, the Cancer Center HUH provided radiation therapy treatments for both pediatric and adult patients with cancer and systemic cancer treatments for most adult patients with cancer. Cancer Center includes a specialist (S)PC clinic that provides outpatient and consultative services for patients at the HUH. SPC services are provided in Finland both by secondary/tertiary and by primary healthcare.

### Data sources and collection

The data collection included gender, diagnoses (ICD-10), outpatient appointments in the Comprehensive Cancer Center HUH, date of the PC decision, visits to the emergency department (ED) in secondary/tertiary care, inpatient episodes and days, date of death and age at death. All data was recorded and extracted in a structured format, except for the date of the PC decision, which was extracted as a combination of Z51.5 diagnosis code or if no diagnosis code was recorded, a mention of PC or EOL care in the free text of physician’s notes. The free text was searched using a few keywords e.g. palliative, terminal care, and Z51.5/Z51.0. The complete list of keywords is provided in Supplementary Material. The date of the PC decision was defined as the date when the Z51.5 diagnosis code was recorded for the first time or the date of the physician’s note where the initiation of palliative treatment was mentioned, unless a different date was specified in the text.

The cancer diagnoses were divided into 15 groups by ICD-10 code: (i) head and neck (HN) cancer (C00-C14, C30-C33), (ii) upper gastrointestinal (GI) cancer (C15-C18, C22-C25), (iii) colorectal cancer (C19-C21), (iv) lung cancer (C34), (v) invasive skin cancers (C43-C44), (vi) breast cancer (C50), (vii) gynecologic cancer (C51-C58), (viii) prostate cancer (C61), (ix) sarcomas (C49), (x) cancers of the urinary tract (C64-C68), (xi) primary central nervous system (CNS) malignancies (C70-C72), (xii) lymphomas (C81-C85), (xiii) myeloma (C90), (xiv) leukemia (C91-C95) and (xv) others. The number of ED visits and hospitalizations were observed 30 and 60 days prior to death.

### Palliative decision and outpatient palliative care clinic visits

The PC decision was defined as a decision to terminate treatment modalities with a curative or life-prolonging intent and to mainly focus on symptom control and QoL. Following the PC decision, a short course of palliative radiotherapy for symptom control was still offered as needed.

The treating oncologists are responsible for making the palliative care decision (PCD). The decision can be made at any timepoint, when the oncologist assesses that the treatments cause more harm than benefits for the patient, there are no treatments left the patient would benefit from, or the patient him- or herself does not want to begin with or continue with the treatments. The timing of the decision can occur both before or after the visit to a PC clinic.

The timing of the outpatient PC clinic visit was determined as follows: (1) integrated visit (2) non-integrated visit and (3) no visit. Outpatient PC clinic visit was defined as an integrated if it was started alongside the usual oncological care i.e. while the patient was still receiving disease modifying treatments with an intent to prolong survival. A non-integrated visit was considered as a first visit following the termination of disease modifying treatments.

### Statistical analysis

All statistics were performed using IBM-SPSS version 29 (IBM Corp, Armonk, NY, USA). Descriptive statistics were reported means with standard deviations (SD) and medians with interquartile ranges (IQR). For categorical data, Pearson’s chi-squared test was used. Differences in median ED visits, inpatient episodes and days between groups were analyzed by Mann-Whitney U test. A p-value < 0.05 was considered as statistically significant.

## Results

The cohort comprised 3744 patients with a mean age of 71 years (SD 11.9) and 1854 (49.5%) were male. In total, 2151 (57.4%) patients visited the outpatient PC clinic during their illness and for 1207 (32.2%) the visit was an integrated visit. The demographics factors according to the timing of the outpatient PC clinic visit are presented in Table 1.

**Table 1:** Demographic data according to the timing of the first palliative care unit visit

	All patients	Integrated outpatient PC clinic visit	Non-Integrated outpatient PC clinic visit	No outpatient PC clinic visit
Number of patients, n (%)	3744 (100)	1207 (32.2)	944 (25.2)	1593 (42.5)
Mean age at death (SD)	71.1 (11.9)	69.4 (11.9)	73.5 (11.5)	71.1 (11.9)
Gender				
Male	1854 (49.5)	630 (34.0)	451 (24.3)	773 (41.7)
Female	1890 (50.5)	577 (30.5)	493 (26.1)	820 (43.4)
Cancer diagnoses, n (%)				
Upper gastrointestinal cancer	961 (20.6)	280 (36.0)	297 (38.2)	201 (25.8)
Lung cancer	512 (13.7)	155 (30.3)	90 (17.6)	267 (52.1)
Breast cancer	436 (11.6)	127 (29.1)	53 (12.2)	256 (58.7)
Colorectal cancer	414 (11.1)	155 (37.4)	109 (26.3)	150 (36.2)
Prostate cancer	289 (7.7)	109 (37.7)	57 (19.7)	123 (42.6)
Gynecologic cancer	272 (7.3)	68 (25.0)	125 (46.0)	79 (29.0)
Cancer of the urinary tract	180 (4.8)	62 (34.4)	41 (22.8)	77 (42.8)
Lymphomas	167 (4.5)	47 (28.1)	20 (12.0)	100 (59.9)
Invasive skin cancers	130 (3.5)	38 (29.2)	33 (25.4)	59 (45.4)
Primary CNS malignancies	123 (3.3)	26 (21.1)	18 (14.6)	79 (64.2)
Head and neck cancer	98 (2.6)	46 (46.9)	22 (22.4)	30 (30.6)
Sarcomas	68 (1.8)	17 (25.0)	12 (17.6)	39 (57.4)
Leukemia	53 (1.4)	10 (18.9)	9 (17.0)	34 (64.2)
Myeloma	45 (1.2)	10 (22.2)	8 (17.8)	27 (60.0)
Other	179 (4.8)	57 (31.8)	50 (27.9)	72 (40.2)

*Abbreviation*: *n* number of patients, *CNS* central nervous system, *SD* standard deviation, *PC* palliative care

The highest access to outpatient PC clinic was among patients with upper GI cancer (74.2%), followed by gynecologic cancer (71%) and HN cancer (69.3%), whereas patients with primary CNS malignancies had the lowest access (35.7%). Integrated PC was most prevalent among patients with HN cancer (46.9%), prostate cancer (37.7%), followed by patients with colorectal cancer (37.4%) and upper GI cancer (36.0%) (Table 1).

### Timing of outpatient PC clinic visit and PC decision

The median time from the first PC visit to death among integrated PC group was significantly longer compared to the non-integrated PC group (129 vs. 55 days; *p* < 0.001) (Table 2). The median number of outpatient PC clinic visits was also higher in the integrated group than in the non-integrated group (4 (IQR 6) vs. 2 (IQR 3); *p* < 0.001).

**Table 2:** The correlation with the timing of the first palliative care unit visit and timing of the PC decision

	All patients	Integrated outpatient PC clinic visit	Non-integrated outpatient PC clinic visit	No outpatient PC clinic visit	*p*-value
Number of patients, n (%)	3744 (100)	1207 (32.2)	944 (25.2)	1593 (42.5)	
Number of PCDs (%)	2780 (74.3)	1046 (86.7) ^a^	831 (88.0) ^b^	895 (56.2) ^c^	0.345^ab^ **< 0.001** ^ac^ **< 0.001** ^bc^
Median time in days between the PCD and death (IQR)	45.5 (99)	61.5 (118) ^a^	58 (117) ^b^	24 (57)^c^	0.405^ab^ **< 0.001** ^ac^ **< 0.001** ^bc^
Median time in days from the first outpatient PC clinic visit to death (IQR)	92 (156)	129 (182) ^a^	55 (116) ^b^	-	**< 0.001** ^ab^

*Abbreviation*: *n* number of patients, *PC* palliative care, *PCD* palliative care decision, *IQR* interquartile range. Parameters with an asterix

^a,b,c^ are compared to each other. *P*-values < 0.05 are bolded

The PC decision was made significantly (*p* < 0.001) more often for patients with an integrated or a non-integrated visit to the outpatient PC clinic than for patients with no visit at all (86.7%, 88% and 56.2%, respectively). The time from PC decision to death was longest in the integrated PC group and shortest with patients who had no PC visit at all (Table 2).

### The association of outpatient PC clinic visits with the use of hospital services

Patients who had a PC visit, either integrated or non-integrated, were less likely to be hospitalized during the last 30 days of life compared to patients who had no outpatient PC clinic visit (36.5% vs. 43.6%; *p* < 0.001 and 35.6% vs. 43.6%; *p* < 0.001, respectively). No differences were found between integrated and non-integrated PC groups. The results were similar within the last 60 days prior to death (Table 3).

**Table 3:** Utilization of emergency department and secondary health care during the last 60 and 30 days prior to death according to the timing of the first palliative care unit visit

	All patients	Integrated outpatient PC clinic visit	Non-integrated outpatient PC clinic visit	No outpatient PC clinic visit	*p*-value
Number of patients, n (%)	3744 (100)	1207 (32.2)	944 (25.2)	1593 (42.5)	
Last 60 days prior to death					
Patients with ED visits (%)	2005 (53.6)	645 (53.4) ^a^	509 (53.9) ^b^	851 (53.4) ^c^	0.824^ab^ 0.993^ac^ 0.808^bc^
Median number of ED visits (IQR)	1 (1)	1 (1) ^a^	1 (1) ^b^	1 (2) ^c^	0.644^ab^ 0.773^ac^ 0.903^bc^
Patients with hospitalizations (%)	1931 (51.6)	594 (49.2) ^a^	450 (47.7) ^b^	887 (55.7) ^c^	0.477^ab^ **< 0.001** ^ac^ **< 0.001** ^bc^
Median number of hospitalizations (IQR)	1 (2)	0 (1) ^a^	0 (0–7) ^b^	1 (2) ^c^	0.742^ab^ **< 0.001** ^ac^ **< 0.001** ^bc^
Median number of inpatient days (IQR)	1 (8)	0 (7) ^a^	0 (6) ^b^	2 (9) ^c^	0.338^ab^ **< 0.001** ^ac^ **< 0.001** ^b^
Last 30 days prior to death					
Patients with ED visits (%)	1450 (38.7)	456 (37.8) ^a^	352 (37.3) ^b^	642 (40.3) ^c^	0.815^ab^ 0.176^ac^ 0.133^bc^
Median number of ED visits (IQR)	0 (1)	0 (1) ^a^	0 (1) ^b^	0 (1) ^c^	0.814^ab^ 0.105^ac^ 0.081^bc^
Patients with hospitalizations (%)	1471 (39.3)	441 (36.5) ^a^	336 (35.6) ^b^	694 (43.6) ^c^	0.651^ab^ **< 0.001** ^ac^ **< 0.001** ^bc^
Median number of hospitalizations (IQR)	0 (1)	0 (7) ^a^	0 (6) ^b^	0 (9) ^c^	0.724^ab^ **< 0.001** ^ac^ **< 0.001** ^bc^
Median number of inpatient days (IQR)	0 (4)	0 (4) ^a^	0 (3) ^b^	0 (6) ^c^	0.612^ab^ **< 0.001** ^ac^ **< 0.001** ^bc^

*Abbreviation*: *n* number of patients, *IQR* interquartile range, *PC* palliative care, *ED* emergency department. Parameters with an asterix

^a,b,c^ are compared to each other. *P*-values < 0.05 are bolded

In addition, compared to patients who did not visit the outpatient PC clinic, those who had a PC visit had a significantly (*p* < 0.001) lower number of inpatient days and lower number of hospitalizations during the last 30 and 60 days prior to death. No significant differences were observed between integrated and non-integrated PC groups (Table 3).

During the last 60 and 30 days of life, no statistically significant difference was found in the number of ED visits between integrated, non-integrated or no outpatient PC clinic visit groups. (Table 3).

### Anticancer therapies during the last month of life

Total of 5.8% of the patients had intravenous (i.v.) chemotherapy during the last 30 days prior to death. Patients with no outpatient PC clinic visit at all were more likely to receive chemotherapy than patients in the other groups. 76 patients (6.3%) in the integrated PC group, 14 patients (1.5%) in the non-integrated PC group and 126 patients (7.9%) in no PC group received i.v. chemotherapy during the last month of life. In total, 8.6% received palliative radiation during the last month of life. No significant differences were observed between the groups.

## Discussion

In the present study more than half of patients with cancer treated in tertiary cancer center were referred to hospital outpatient PC clinic and approximately in one third the first visit was considered as integrated i.e. it was conducted while the patient was still receiving active oncological treatments. Patients who had a PC clinic visit were less likely to be hospitalized during the last 60 and 30 days of life compared to patients who had no PC clinic visit at all despite of the timing of the first PC visit. However, no effect on ED visits was found. The PC decision i.e. decision to terminate life-prolonging anticancer treatments, was made relatively early, thus two months prior to death, in the disease trajectory if there was a visit to outpatient PC clinic.

Patients in our cohort had relatively good access to outpatient PC clinic as nearly 60% of patients visited outpatient PC clinic at some point during their illness trajectory. For comparison, in a Finnish registry-based study with 6010 patients with cancer and treated in tertiary hospitals a outpatient PC clinic was visited by 37% of the patients a median 112 days before death [16]. It should be noted however that the patient cohort of that study was from 2013 to 2014 showing that the PC pathway has evolved in recent years. A more recent Finnish nationwide register-based study, including all adults (*n* = 38 540) who died from non-communicable life-limiting diseases in 2019, reported that 30% of patients with cancer had a PC contact [17]. Adsersen et al. [18] found in a Danish nationwide register-based study with data of 44 548 patients with cancer that only about 40% of patients were admitted to SPC. We can cautiously say that the findings in our present study with tertiary hospital data are quite encouraging even by international standards [18].

Integrated PC during active oncological treatments was implemented for one third of the patients in our cohort. It would be essential that a larger proportion of patients should have contact with the PC outpatient clinic at an earlier stage, but based on the literature, large-scale implementation of early PC, has not been adopted despite its well-documented benefits [19–22].

Significant differences were observed between tumor types regarding access to the PC outpatient clinic. The access to PC was highest in patients with upper GI cancer, gynecologic cancer and HN cancer. These results are relatively favorable, for example, compared to a previous cohort of 960 patients with non-curative upper GI cancers, only 55% were referred to SPC clinic [23]. Similarly, among 221 patients with pancreatic cancer 49% visited PC unit [24] and among 8297 patients with ovarian cancer only 32% received PC in the last 3 months of life [25]. Unfortunately, our study confirmed the previous findings that patients with primary CNS malignancies are less likely to have outpatient PC clinic contact, as in the data of Dulley et al., which included 37,500 patients, only 15% of the patients were referred to a PC clinic [26]. In our data, approximately one third of patients with CNS malignancies visited a outpatient PC clinic.

In the present cohort, use of anticancer treatments at the EOL was modest. Chemotherapy was used only in 6% of the patients during the last month of life. Ma et al. [27] reported that 12% of patients with cancer received chemotherapy in the last 14 days before death. In the same recent meta-analyses 24% received chemotherapy in the last 30 days and 13% started a new chemotherapy regimen in the last 30 days of life. Palliative radiotherapy was given in 9% of the patients in our cohort during the last months of life, which is in line with previous studies reporting that 7–10% of patients with cancer received radiotherapy in the last 30 days of life [27, 28]. Integrated PC has reduced aggressive care at EOL over best supportive care as in the landmark study of Temel et al. aggressive treatments during the last 14 days reduced from 50% to 30% [6]. Similarly, Maltoni et al. found that initiating PC early in a cohort of 209 patients with locally advanced or metastatic pancreatic cancer was associated with less aggressive treatment approaches at the EOL [29]. The small difference observed between the groups in our study may reflect the overall decrease in the use of cancer treatments during the final month of life.

In line with our previous studies, there seemed to be an association between timing of PC decision i.e. withholding of life-prolonging anticancer treatments and outpatient PC clinic contact [16, 30, 31]. 964 patients did not have a PC decision, and only 216 patients received i.v. chemotherapy during their last month of life. 321 patients received palliative radiotherapy. This means that even though a PC decision had not been made, not all of those patients received oncological treatment at the end of life. Patients who had visit to outpatient PC clinic had PC decisions made significantly more often than patients with no outpatient PC clinic visit at all, and even slightly earlier if the PC visit was started already during anticancer treatment compared to the PC contact starting after terminating the cancer treatments. Despite this, it seems that the PC team plays a smaller role than expected in PC decision making even though it is one of the indicators to refer to PC [32]. Instead, the individual oncologist seems to be responsible for the decisions of withholding the anticancer treatments and referral to PC if there are no systematic indicators and triggers for integrated PC.

In our cohort, both hospitalizations and inpatient days at the EOL were significantly lower among patients who had at least one visit to the outpatient PC clinic compared to those who had no visits at all. However, the association between outpatient PC clinic utilization and ED visits was weak. Nevertheless, more than one third of all patients in the study were hospitalized and visited ED during the last month of life.

De Man et al. have shown that receiving active oncological treatments during EOL phase is strongly associated with increased ED and intensive care unit contacts [33]. For example, among 383 patients with incurable cancer who received systemic anticancer therapy within their last 30 days of life, hospitalization and in-hospital deaths were more likely [34]. In another study of 340 patients with metastatic breast cancer, PC intervention initiated more than 30 days before death was associated with reduced chemotherapy use in the last month [35].

There are several plausible explanations for the limited impact of outpatient PC clinic visits on ED utilization in this cohort. First, the timing of PC involvement was relatively late: the median time from the first PC visit to death was only 92 days. Even in integrated PC group, it was approximately four months before death. The number of visits was relatively low: a median of 4 visits in the integrated PC group and 2 visits in the non-integrated PC group. In comparison, Haltia et al. reported better outcomes with earlier PC involvement (median 112 days before death) with a median of 3 visits [16]. They found a clearer reduction in ED visits among patients who had visited the outpatient PC clinic.

Beyond timing and continuity, effective reduction of ED visits at the EOL also requires timely access to acute care. While some ED visits are unavoidable due to cancer-related complications requiring emergency treatment, others might be preventable with rapid-response PC services. During the study in Finland, the readiness to arrange acute PC was limited e.g. PC physician support was not available during on-call hours in home hospitals, causing unnecessary ED visits and unwanted hospital admissions.

In previous randomized controlled trial (RCT) studies integrated PC has improved QoL, symptom control and satisfaction of patients and caregivers to care [29, 36]. In addition, access to PC services has improved. In our real-life cohort, we investigated the association of PC to acute hospital service utilisation comparing integrated, non-integrated and no PC. Despite of the benefit of PC over no PC, we could not demonstrate major advantages of integrated over non-integrated PC on acute health care utilization at the EOL.

A possible explanation for the lack of difference in the present cohort could be that it was a real-life cohort. In real life PC services were limited and integrated PC started later that what has been seen in various RCTs [37]. PC interventions were sparse and partly implemented by telephone. Noteworthily, in the present cohort, only 6% of the patients had i.v. chemotherapy during the last months of life, so there was no room for improvement. We have previously demonstrated that access to PC at least 30 days before death significantly reduces the acute hospital utilization [16, 31, 38, 39]. Thus, hypothetically, early access to PC even as a non- integrated way could be sufficient for reducing acute hospital utilization during the last months of life. The effect of integrated PC on health care utilization is presumably seen in the earlier phase of the disease trajectory, in advance care planning and decision making including timely PC decision and termination of anticancer treatments without forgetting QoL advantage. To prove this hypothesis further RCT studies are needed.

The limitations of this study are its retrospective study design and that we only had data on outpatient PC clinic visits in tertiary healthcare. Data on other sites offering PC at the EOL were missing. Some patients have been referred directly to primary healthcare SPC clinics without a visit to tertiary center outpatient PC clinic. Thus, the total number of patients having contact to any SPC clinic is even higher than presented here. In addition, we only had information about iv chemotherapy as no data on oral chemotherapy, endocrine or biological treatments was available underestimating the use of anticancer treatments at the EOL. The strength of our research is its real-life data representing all the patients treated at university hospital and deceased thereafter. In addition, the data on the use of hospital services is mostly quite comprehensive and robust.

## Conclusions

The access to SPC among patients with cancer has improved over the last years and the contact starts relatively often already alongside with the active oncological care. PC visits, both integrated and non-integrated, seem to be associated with fewer hospitalizations and inpatient days in secondary healthcare at the EOL.

## Supplementary Information

Supplementary Material 1.

## Data Availability

The data generated for the current study are not publicly available as the data is a part of the larger dataset owned by Helsinki University Hospital. Data are, however, available from the principal author Tiina Saarto, tiina.saarto@hus.fi upon reasonable request and with permission of Helsinki University Hospital.

## Abbreviations

CNS: Central nervous system
ED: Emergency department
EOL: End of life
GI: Gastrointestinal
HN: Head and neck
HUH: Helsinki University Hospital
i.v.: Intravenous
PC: Palliative care
QoL: Quality of life
RCT: Randomized controlled trial
SD: Standard deviation
SPC: Specialist palliative care

## References

[1] Ferlay J, Ervik M, Lam F, Colombet M, Mery L, Piñeros M, et al. Global cancer observatory: cancer today. Lyon: International Agency for Research on Cancer; 2020.

[2] Siegel RL, Miller KD, Fedewa SA, Ahnen DJ, Meester RGS, et al. Colorectal cancer statistics, 2017. CA Cancer J Clin. 2017;67:177–93. 28248415 10.3322/caac.21395

[3] Ervik M, Lam F, Laversanne M, Ferlay J, Bray F. Global cancer observatory: cancer over time. Lyon, France: International Agency for Research on Cancer; 2021.

[4] Kroenke K, Zhong X, Theobald D, Wu J, Tu W, Carpenter J. Somatic symptoms in patients with cancer experiencing pain or depression: prevalence, disability, and health care use. Arch Intern Med. 2010;170:1686–94. 20937930 10.1001/archinternmed.2010.337 PMC3174492

[5] Zimmermann C, Swami N, Krzyzanowska M, Hannon B, Leighl N, Oza A, et al. Early palliative care for patients with advanced cancer: a cluster-randomised controlled trial. Lancet. 2014;383:1721–30. 24559581 10.1016/S0140-6736(13)62416-2

[6] Temel JS, Greer JA, Muzikansky A, Gallagher ER, Admane S, Jackson VA, et al. Early palliative care for patients with metastatic non-small-cell lung cancer. N Engl J Med. 2010;363:733–42. 20818875 10.1056/NEJMoa1000678

[7] Lammers A, Slatore CG, Fromme EK, Vranas KC, Sullivan DR. Association of early palliative care with chemotherapy intensity in patients with advanced stage lung cancer: A National cohort study. J Thorac Oncol. 2019;14:176–83. 30336324 10.1016/j.jtho.2018.09.029 PMC6348039

[8] Woldie I, Elfiki T, Kulkarni S, Springer C, McArthur E, Freeman N. Chemotherapy during the last 30 days of life and the role of palliative care referral, a single center experience. BMC Palliat Care. 2022;21:20. 35125092 10.1186/s12904-022-00910-x PMC8819957

[9] Le NS, Zeybek A, Hackner K, Gottsauner-Wolf S, Groissenberger I, Jutz F, et al. Systemic anticancer therapy near the end of life: an analysis of factors influencing treatment in advanced tumor disease. ESMO Open. 2024;9:103683. 39214050 10.1016/j.esmoop.2024.103683 PMC11402042

[10] Zhang Z, Lovell A, Subramaniam D, Hinyard L. The impact of palliative care consultation on aggressive medical interventions in end-of-life among patients with metastatic breast cancer: insight from the U.S National patient sample. J Palliat Care. 2025;40:8–17. 38748597 10.1177/08258597241253933

[11] Blackhall LJ, Read P, Stukenborg G, Dillon P, Barclay J, Romano A, et al. CARE track for advanced cancer: impact and timing of an outpatient palliative care clinic. J Palliat Med. 2016;19:57–63. 26624851 10.1089/jpm.2015.0272 PMC4692130

[12] Ho TH, Barbera L, Saskin R, Lu H, Neville BA, Earle CC. Trends in the aggressiveness of end-of-life cancer care in the universal health care system of Ontario, Canada. J Clin Oncol. 2011;29:1587–91. 21402603 10.1200/JCO.2010.31.9897 PMC3082976

[13] Warren JL, Barbera L, Bremner KE, Yabroff KR, Hoch JS, Barret MJ, et al. End-of-life care for lung cancer patients in the united States and Ontario. J Natl Cancer Inst. 2011;103:853–62. 21593012 10.1093/jnci/djr145 PMC3115676

[14] Riley G, Lubitz J. Long-term trends in medicare payments in the last year of life. Health Serv Res. 2010;45:565–76. 20148984 10.1111/j.1475-6773.2010.01082.x PMC2838161

[15] Monnery D, Droney J. Early palliative care and its impact on end of life care. Curr Opin Support Palliat Care. 2024;18:230–4. 39351632 10.1097/SPC.0000000000000729

[16] Haltia O, Vesinurm M, Leskelä RL, Rahko E, Tyynelä-Korhonen K, Lehti JT, et al. The effect of palliative outpatient units on resource use for cancer patients in Finland. Acta Oncol. 2023;62:1118–23. 37535611 10.1080/0284186X.2023.2241988

[17] Ahtiluoto S, Carpen T, Forsius P, Nuutinen M, Nåhls NS, Kitti P., et al. Impact of specialist palliative care on utilization of healthcare and social services at the end-of-life: a nationwide register-based cohort study. Eur J Public Health. 2025;35:828–34. 10.1093/eurpub/ckaf044. 10.1093/eurpub/ckaf044 PMC12529283 40434100

[18] Adsersen M, Thygesen LC, Jensen AB, Neergaard MA, Sjogren P, Groenvold M. Is admittance to specialised palliative care among cancer patients related to sex, age and cancer diagnosis? A nation-wide study from the Danish palliative care database (DPD). BMC Palliat Care. 2017;16:21. 28330507 10.1186/s12904-017-0194-z PMC5363002

[19] Wentlandt K, Krzyzanowska MK, Swami N, Rodin GM, Le LW, Zimmermann C. Referral practices of oncologists to specialized palliative care. J Cin Oncol. 2012;30:4380–6. 10.1200/JCO.2012.44.0248 23109708

[20] Smith CB, Nelson JE, Berman AR, Powell CA, Fleischman J, Salazar-Schicchi, et al. Lung cancer physicians’ referral practices for palliate care consultation. Ann Oncol. 2012;23:382–7. 21804051 10.1093/annonc/mdr345 PMC3265546

[21] Feld E, Singhi EK, Phillips S, Huang L, Shyr Y, Horn L. Palliative care referrals for advanced Non–small-cell lung cancer (NSCLC): patient and provider attitudes and practices. Clin Lung Cancer. 2019;20:e291–8. 30862422 10.1016/j.cllc.2019.02.002

[22] Paiva C, de Lourdes Gonçalves F, Salete de Angelis Nascimento M, Coutinho Zago F, Donizete Costa E, Beraldo Ciorlia J, et al. Missed opportunities of integration of palliative care: Frequency, Causes, and profile of missed visits in an oncologic palliative care outpatient unit. J Pain Symptom Manage. 2020;59:1067–73. 31988019 10.1016/j.jpainsymman.2020.01.005

[23] Boland E, Tay K, Khamis A, Murtagh F. Patterns of acute hospital and specialist palliative care use among people with non-curative upper Gastrointestinal cancer. Support Care Cancer. 2024;32:432. 38874678 10.1007/s00520-024-08624-x PMC11178555

[24] Miinalainen S, Rissanen A, Leskelä RL, Saarto T, Hirvonen O, Anttonen A. Effect of palliative care decision on use of hospital services in pancreatic cancer patients: A retrospective study. Antica Res. 2022;42:5457–63. 10.21873/anticanres.16050 36288858

[25] Mah J, Ramirez D, Schnarr K, Eiriksson L, Gayowsky A, Seow H. Timing of palliative care, end-of-life quality indicators, and health resource utilization. JAMA Netw Open. 2024;7:e2440977. 39466244 10.1001/jamanetworkopen.2024.40977 PMC11519754

[26] Dullea JT, Vasan V, Devarajan A, Ali A, Nichols N, Chaluts D, et al. Utilization of palliative care services among patients with malignant brain tumors: an analysis of the National inpatient sample (2016–2019). Neurosurg. 2023;93:419–26. 10.1227/neu.0000000000002428 36867460

[27] Ma Z, Huangqianyu L, Yi Z, Lan Z, Guo H, Yichen Z, et al. Prevalence of aggressive care among patients with cancer near end of life: a systematic review and meta-analysis. eClinicalMedicine. 2024;71:102561. 38549585 10.1016/j.eclinm.2024.102561 PMC10972834

[28] Park KR, Chang G, Yolanda D, Jay L, Suresh R, Eduardo B, et al. Palliative radiation therapy in the last 30 days of life: a systematic review. Radiother Oncol. 2017;125:193–99. 29050955 10.1016/j.radonc.2017.09.016

[29] Maltoni M, Scarpi E, Dall’Agata M, Schiavon S, Biasini C, Codecà C, et al. Systematic versus On-Demand early palliative care: A randomised clinical trial assessing quality of care and treatment aggressiveness near the end of life. Eur J Cancer. 2016;69:110–18. 27821313 10.1016/j.ejca.2016.10.004

[30] Hirvonen O, Leskelä RL, Grönholm L, Haltia O, Voltti S, Tyynelä-Korhonen K, et al. The impact of the duration of the palliative care period on cancer patients with regard to the use of hospital services and the place of death: a retrospective cohort study. BMC Palliat Care. 2020;19:37. 32209075 10.1186/s12904-020-00547-8 PMC7093948

[31] Nåhls N, Leskelä RL, Saarto T, Hirvonen O, Anttonen A. Effect of palliative care decisions making on hospital service use at end-of-life in patients with malignant brain tumors: a retrospective study. BMC Palliat Care. 2023;22:39. 37032344 10.1186/s12904-023-01154-z PMC10084612

[32] Hui D, Moei M, Watanabe SM, Caraceni A, Strasser F, Saarto T, et al. Referral criteria for outpatient specialty palliative cancer care: an international consensus. Lancet Oncol. 2016;17:e552–9. 27924753 10.1016/S1470-2045(16)30577-0

[33] de Man Y, Atsma F, Oosterveld-Vlug M, Brom L, Onwuteaka-Philipsen B, Westert GP, et al. The intensity of hospital care utilization by Dutch patients with lung or colorectal cancer in their final months of life. Cancer Control. 2019;26:1–9. 10.1177/1073274819846574 PMC6552371 31159571

[34] Singh J, Stensvold A, Turzer M, Grov E. Anticancer therapy at end-of-life: A retrospective cohort study. Acta Oncol. 2024;63:313–21. 38716486 10.2340/1651-226X.2024.22139 PMC11332458

[35] Lucchi E, Berger F, Milder M, Commer J-M, Morin S, Capodano G, et al. Palliative care interventions and end-of-life care for patients with metastatic breast cancer: a multicentre analysis. Oncologist. 2024;29:e708–15. 38387031 10.1093/oncolo/oyae023 PMC11067792

[36] Vanbutsele G, Pardon K, Van Belle S, Surmont V, De Laat M, Colman R, et al. Effect of early and systematic integration of palliative care in patients with advanced cancer: A randomised controlled trial. Lancet Oncol. 2018;19:394–404. 29402701 10.1016/S1470-2045(18)30060-3

[37] Gaertner J, Siemens W, Meerpohl JJ, Antes G, Meffert C, Xander C, et al. Effect of specialist palliative care services on quality of life in adults with advanced incurable illness in Hospital, Hospice, or community settings: systematic review and Meta-Analysis. BMJ. 2017;357:j2925. 28676557 10.1136/bmj.j2925 PMC5496011

[38] Kitti P, Anttonen A, Leskelä RL, Saarto T. End-of-life care of patients with esophageal or gastric cancer: decision making and the goal of care. Acta Oncol. 2022;61:1173–8. 36005550 10.1080/0284186X.2022.2114379

[39] Kitti P, Anttonen A, Nuutinen M, Carpen T, Saarto T. Specialist palliative care is associated with reduced healthcare utilization in patients with advanced esophageal and gastric cancer: a nationwide register-based study. Support Care Cancer. 2025;33:540. 40471285 10.1007/s00520-025-09587-3 PMC12141135

