# Supplementary material for: The association of the timing of outpatient palliative care clinic visit on the utilization of hospital services and decision making at the end of life in patients with cancer – a retrospective cohort study
Source: BMC Cancer. 2025 Nov 18;25:1777. doi: 10.1186/s12885-025-15242-1 (PMC12625559; doi:10.1186/s12885-025-15242-1)
Supplement: Supplementary file 1 — Supplementary Material 1. [file 12885_2025_15242_MOESM1_ESM.docx]

Supplementary Material

The complete list of keywords

oireenmuk* palliative

palliat* palliative

saattohoi* terminal care

kuoleva dying

hoitolin* care line

hoitotes* care will

tahdonilm* expression of will

elvyt* resuscitation

dnr

dnar

Z51/ Z51.5

loppuvaih* final stage

perushoi* primary care

termin* terminal

tehohoi* intensive care

ER* DNR
